# Transcriptional Gene Silencing (TGS) via the RNAi Machinery in HIV-1 Infections

**DOI:** 10.3390/biology1020339

**Published:** 2012-08-24

**Authors:** Gavin C Sampey, Irene Guendel, Ravi Das, Elizabeth Jaworski, Zachary Klase, Aarthi Narayanan, Kylene Kehn-Hall, Fatah Kashanchi

**Affiliations:** 1 National Center for Biodefense and Infectious Disease, School of Systems Biology, George Mason University, 10900 University Blvd, Manassas, VA 20108, USA; Email: gsampey@gmu.edu (G.C.S.); iguendel@gmu.edu (I.G.); dasravi85@gmail.com (R.D.); emjaws@gmail.com (E.J.); anaraya1@gmu.edu (A.N.); kkehnhal@gmu.edu (K.K.-H.); 2 Molecular Virology Section, Laboratory of Molecular Microbiology, National Institute of Allergy and Infectious Diseases, 9000 Rockville Pike, Bethesda, MD 20810, USA; Email: klaseza@niaid.nih.gov

**Keywords:** HIV, miRNA, RNAi, transcriptional gene silencing, chromatin remodeler

## Abstract

Gene silencing via non-coding RNA, such as siRNA and miRNA, can occur at the transcriptional, post-transcriptional, and translational stages of expression. Transcriptional gene silencing (TGS) involving the RNAi machinery generally occurs through DNA methylation, as well as histone post-translational modifications, and corresponding remodeling of chromatin around the target gene into a heterochromatic state. The mechanism by which mammalian TGS occurs includes the recruitment of RNA-induced initiation of transcriptional gene silencing (RITS) complexes, DNA methyltransferases (DNMTs), and other chromatin remodelers. Additionally, virally infected cells encoding miRNAs have also been shown to manipulate the host cell RNAi machinery to induce TGS at the viral genome, thereby establishing latency. Furthermore, the introduction of exogenous siRNA and shRNA into infected cells that target integrated viral promoters can greatly suppress viral transcription via TGS. Here we examine the latest findings regarding mammalian TGS, specifically focusing on HIV-1 infected cells, and discuss future avenues of exploration in this field.

## 1. Introduction

As of 2009, the UNAIDS report on the global acquired immunodeficiency syndrome (AIDS) epidemic indicated 33.3 million people living with human immunodeficiency virus (HIV). An additional 2.6 million new HIV infections were calculated to take place annually, with the vast majority of these cases occurring in developing countries and greater than 50% in women [1]. Current projections suggest continued increase in the number of people infected with a possible resurgence of this pandemic in high-income countries, such as the United States. One possible reason for this resurgence is the problems associated with highly active anti-retroviral therapies (HAART) in HIV-1 treatment. HAART, a multi-drug therapy given to AIDS patients, has proven to be effective at lowering viral loads (<50 copies/mL in plasma) and improving an individual’s immune response to HIV-1 infection. However, limitations and concerns regarding cost, complexity of treatment, and long-term side effects have led to lower adherence to treatment regimens by some patients. Also, the development of drug-resistant mutants, either through a lack of protocol adherence or supervised/structured treatment interruptions (STIs), has increasingly become problematic [2,3]. Due to the persistence and continuous alteration of HIV-1 viral infections, a better understanding of all underlying molecular mechanisms involved in the viral life cycle is critical to battling this pathogen.

Across all living organisms there are various mechanisms by which attenuation or complete silencing of genes can be facilitated. Those gene silencing systems that involve RNA interference (RNAi) components include inhibition at the stages of transcription, post-transcription, and translation. Transcriptional gene silencing (TGS) occurring through RNA complementary to gene promoters was first observed and examined in detail in plant species starting around 2000 [4]. In 2004, studies utilizing *S. pombe* fission yeast first identified several of the components of the RNAi-dependent RITS complex that are required for TGS and postulated a mechanism of action [5,6]. That same year, mammalian species were shown to carry the same molecular mechanism of gene silencing. Specifically, a key study demonstrated TGS in mammalian cell lines utilizing small interfering RNAs (siRNAs) targeted against the promoters of a couple genes of interest [7]. In parallel, another study demonstrated that microRNAs (miRNAs) derived from the *nef* gene of HIV-1 could induce TGS of the integrated viral genome, thereby leading to viral latency [8].

In this review we will first examine the individual molecular and cellular facets that are required for the induction of TGS, specifically the RNAi and chromatin remodeling machinery. We will then delve into the current understanding of TGS and how it applies to HIV-1 infections, as well as explore prospective lines of future inquiry in this field. 

## 2. MicroRNA and the RNAi Molecular Machinery

### 2.1. MicroRNA Biogenesis and the RNAi Machinery

The biogenesis of miRNAs has been very well characterized and described in multiple articles [9,10,11,12,13,14,15]. MicroRNAs are genome encoded RNA hairpin structures that are transcribed by RNA polymerase II (Pol II) as primary transcripts of up to several kilobases in length. Often, many primary transcripts contain multiple hairpin structures in their intronic and/or untranslated regions that are processed by RNase III class enzymes in the nucleus and cytoplasm to yield the final mature miRNA product that measures about 22 bases in length. The mature miRNA products are then incorporated into effector molecular complexes that finally serve as antisense regulators of gene expression.

The primary transcript (pri-miRNA) [16] is processed in the nucleus by the RNase III enzyme Drosha in conjunction with its double-stranded RNA-binding cofactor, DiGeorge syndrome critical region 8 (DGCR8) [17,18,19,20]. Drosha cleaves the miRNA at about 22 base pairs down-stream of the stem-loop structure to generate an approximately 60 nucleotide long pre-miRNA with a 2 nucleotide 3’ overhang. The two nucleotide 3’ overhang in the pre-miRNA is recognized by the exportin-5/Ran GTP complex which then facilitates pre-miRNA export out of the nucleus [21,22]. In the cytoplasm, the pre-miRNA is bound by a second RNase III enzyme Dicer that cleaves the RNA about two helical turns into the hairpin and degrades the terminal loop structure [23,24,25]. Dicer acts in association with the HIV-1 TAR binding protein (TRBP) [26,27] and generates a miRNA duplex of approximately 22 nucleotides with a 2 nucleotide overhang at the 3’ ends of both strands. One strand of this duplex (the “passenger strand”) is degraded while the other (the “guide strand”) is incorporated into the RNA induced silencing complex (RISC) [28]. The catalytic components of RISC are the Argonaute proteins (Ago 1–4), of which Ago2 has been shown to have endonuclease activity and can cleave target mRNAs that show complementarity to the guide strand.

Post-transcriptionally, the RISC complex and the associated miRNA were first found to bind to the 3’ UTR region of the target mRNAs but subsequent studies found targeting of the 5’ UTR and coding regions as well [29,30,31,32,33]. Nucleotides 2–7 of the miRNA, called “the seed”, play an important role in the positioning of the RISC complex and the associated miRNA on the target mRNA [34,35]. The degree of complementarity between the target mRNA and the effector miRNA is a determining factor that decides if the target mRNA is degraded or if it is translationally repressed. Perfect complementarity between the target and miRNA will result in mRNA degradation. However, incomplete complementarity will result in translational repression. 

**Figure 1:**
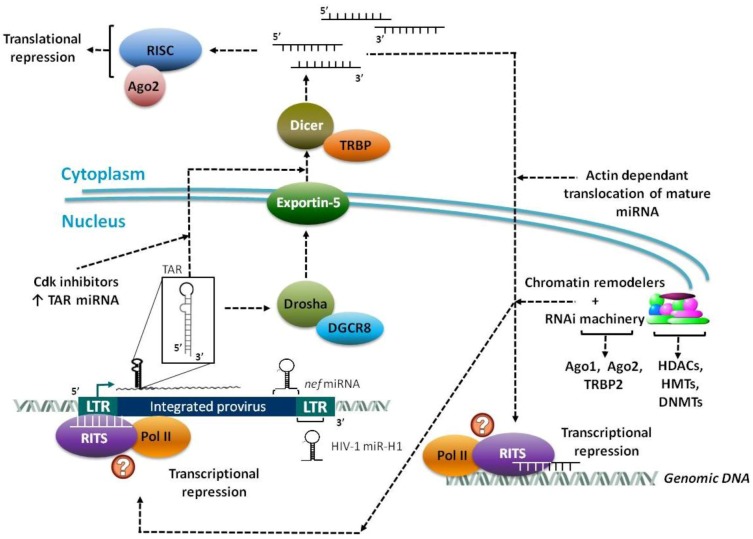
Several pri-miRNAs transcribed from the integrated viral genome are processed by Drosha and DGCR8 into pre-miRNAs. The pre-miRNAs are then exported to the cytoplasm via Exportin 5 where mature miRNAs are generated by Dicer. These miRNAs can either be incorporated into the post-transcriptional repressive RNA induced silencing complex (RISC) complex in the cytoplasm or translocated back into the nucleus in an actin dependent manner. In the nucleus, the miRNAs join the RITS complex that then establish transcriptional gene silencing (TGS) by targeting host genome or integrated viral promoters and rapidly recruiting repressive chromatin remodelers.

In addition to post-transcriptional processing, RNA mediated silencing can also operate at the chromatin level to regulate gene expression. MicroRNAs can associate with the RITS complex and be guided to complementary regions in the chromosomal DNA [36,37]. Following association with such genomic regions, the RITS complex recruits factors, such as histone modifying enzymes, which alter the chromatin structure and induce transcriptional silencing [5,37]. This mechanism of TGS is the primary focus of this review and will be elaborated upon in the later sections (Figure 1).

Alternately, some studies have demonstrated that exogenous siRNAs and endogenous miRNAs that target A:T rich regions of several gene promoters actually activate gene expression [38,39]. The mechanism of action for the observed gene activation was later found to be driven by Ago2 mediated degradation of antisense transcripts that were directing epigenetic repression of the sense strand transcription [40].

While the above-mentioned mechanism is the most commonly observed processing mechanism for generation of miRNAs, several alternate pathways have also been postulated based largely on deep sequencing studies. These studies offer interesting alternatives to the conventional pathway; however, they are largely preliminary and require more extensive validation. A recent review by Yang *et al.* describes in excellent detail, the various alternate mechanisms for miRNA production [15]. 

### 2.2. Virally Encoded miRNAs

The crucial role of miRNAs in gene regulation makes them an obvious target for viruses to hijack in order to regulate viral and host gene expression. Thus, there are significant advantages for viruses that can generate miRNA and exploit the RNAi machinery for host and viral gene regulation. Furthermore, there is ample evidence to support the theory that viruses themselves can generate miRNAs. Firstly, unlike viral proteins, miRNAs are not antigenic as they can avoid the INF/PKR induced pathway, which is normally triggered double stranded RNA (dsRNA) of at least 45 bp in length [41,42]. Additionally, viral miRNAs are able to successfully down-regulate the expression of host gene products that harbor antiviral functionalities. Finally, their small space requirement of around 200-bp of the viral genome is of significant advantage given the tight constraints on viral genome size [41]. Since the original discovery of miRNAs in Epstein-Barr virus (EBV) [43,44], more than 235 viral miRNAs have been identified across all strains examined and listed in the miRNA repository miRBase [45,46].

Viral miRNAs follow the same biogenesis pathways as cellular miRNA (see Section 2.1). There are various techniques for the detection of viral miRNA in an infected cell, most of which begins with bioinformatics analyses to identify stem-loop structures matching pre-miRNA. This is followed by cDNA cloning and high-throughput sequencing of large numbers of the resultant clones [47,48]. These clones are subjected to Northern blot analyses with total cellular RNA, which provides additional confirmation. Massively parallel deep sequencing is another widely used method for the detection of viral miRNA, such as the use of the highly sensitive SOLiD^TM^ 3 Plus System to analyze viral RNA accumulation in HIV-1-infected T lymphocytes. This method detected numerous small RNAs that correspond to the HIV-1 RNA genome [49]. 

#### 2.2.1. Primary Functions of Viral miRNAs

The role of viral miRNAs in regulating host gene expression is well-established. Bioinformatics analysis of the targets of EBV-encoded miRNAs revealed that the cellular transcripts targeted by these miRNAs are over-represented in the genes associated with apoptosis and proliferation, as well as cytokines, signal transduction components, and transcriptional regulators [44]. After high-throughput sequencing and cross-linking immunoprecipitation (HITS-CLIP), Kasandra *et al.* identified mRNA targets of 44 EBV and 310 human miRNAs in Jijoye (Latency III) EBV-transformed B cells. They reported that 1664 human 3'-untranslated regions are targeted by the 12 most abundant EBV miRNAs [50]. Moreover, Herpes Simplex Virus-encoded miRNAs were found to target transcripts involved in apoptosis [51]. MicroRNAs purported to be generated from HIV-1 have been predicted to target several cellular genes, such as p21 activated protein kinase Pak2, extracellular signal regulator kinase 8, MHC class II, IкB kinase-beta, proteasome 26S subunit, CD68 antigen, and tyrosine kinase pp60c-src [52].

Another key reason for viruses to encode miRNA is to self-regulate viral transcripts and, thereby, generate or fine-tune arrays of viral protein expression. Some of the known examples of viral mRNA targets include transcripts that are transcribed antisense to the viral miRNA precursor [44,47,48]. Hence, the fate of these transcripts upon binding to the perfectly matched miRNA can be their degradation by Ago2-containing RISC complexes, if they are co-expressed during the virus life cycle. For instance, Pfeffer *et al.* described that EBV miR-BART2 exhibits perfect complementarity to the 3’ UTR of the mRNA for the EBV DNA polymerase BALF5, which is transcribed antisense to miR-BART2 [44]. Also, another study identified a miRNA stem-loop precursor in the genome of polyoma virus SV40, which produces two miRNAs that are expressed late during viral infection, miR-S15p and miR-S1-3p. Their study concluded that both SV40 derived miRNAs are perfectly complementary to early mRNA transcribed antisense to the pre-miRNA precursors, and aid in the cleavage of these early transcripts, which code for the large and small T antigens [47]. Additional studies have also demonstrated recognition of viral transcripts by viral miRNAs, but through imperfect 3’ UTR complementarity [53,54,55]. As previously mentioned, miRNAs also have the capability of inducing TGS through the RITS complex and associated recruitment of chromatin-remodeling factors [7,36,37,56,57,58,59]. For instance, histone deacetylase-1 (HDAC-1) is recruited to the HIV-1 long terminal repeat (LTR) in presence of either transactivation response (TAR) element specific siRNA, or TAR-derived viral miRNA, which is capable of silencing gene expression through TGS [60]. The establishment of TGS and viral latency via HIV-1 derived miRNAs will be examined in more detail in a subsequent section.

#### 2.2.2. MicroRNAs from DNA and RNA Viruses

DNA viruses examined to date including human adenovirus, virescens ascovirus (HvAc), a baculovirus, several members of the polyoma virus family, as well as all herpes viruses, have been found to encode miRNAs [47,61,62,63,64,65,66,67,68,69]. In addition, being nuclear DNA viruses, they have easy access to Drosha and DGCR8, which are localized to the nucleus, and are required for the initial pre-miRNA excision event as previously discussed. Most of the miRNAs encoded by these viruses may aid in the establishment of latency, lytic switch, immune evasion, cell survival and proliferation [70]. 

Despite considerable controversy over whether or not RNA viruses encode miRNAs [71], there have been several studies that have shown that several RNA viruses do indeed encode for miRNA, which include HIV-1, influenza virus, and bovine leukemia virus (BLV) [49,70,72,73,74,75,76]. In one study done by Yeung *et al.*, they were able to identify several small non-coding RNAs (ncRNAs) of around 18-nt in HIV-1 infected MT-4 T-cells. The findings also show the association of these small RNAs with Ago2, indicating its possible function in the cellular RNAi machinery for targeting HIV-1 [77]. Furthermore, it has also been reported that the TAR ncRNAs were the most abundant followed by the RRE and *nef* ncRNAs. Other studies have also identified the *pol*, *gag* and *env* regions of HIV-1 as likely viral miRNA-encoding candidates [49]. Similarly, other RNA viruses, such as influenza virus, have also been reported to generate small viral RNAs (svRNAs) of between 22 to 27-nt nucleotides in length. The depletion of the svRNAs in infected cells results in a dramatic loss of other viral RNA in a segment-specific manner, indicating their function in the regulation of viral transcription [75]. More recently, it was demonstrated that the BLV genome contains a cluster of miRNAs that are transcribed by RNA polymerase III. Moreover, one of the identified BLV encoded miRNAs was highly homologous to a host oncogenic miRNA both of which give rise to a similar type of B-cell tumor when over-expressed [73]. As previously mentioned, miRNAs generated from HIV-1 have been predicted to target a wide range of cellular genes and, therefore, could drastically affect the infected host-cell stasis [49,52].

#### 2.2.3. HIV-1 Derived miRNAs

Numerous studies have found that there are several miRNAs derived from the integrated HIV-1 genome, including from the TAR element, 3’ LTR, and *nef* gene. The TAR element is a RNA sequence located in the R region of the HIV-1 LTR, which is present at both 5’ and 3’ ends of the HIV-1 transcripts [78,79]. TAR forms a stem-bulge-loop RNA structure that is recognized by the HIV-1 Tat protein, which recruits the cellular factor P-TEFb, allowing for efficient viral transcription [80,81]. Structure and interactome analysis of TAR element suggest that it could potentially give rise to a miRNA. The presence of a hairpin structure, a required structure of a Dicer substrate, in the TAR element suggests its association with the Dicer [52]. Moreover, the interaction of TAR with TRBP and Dicer, which are among the well-characterized proteins identified as crucial components of RISC [82], also strongly supports the possibility of TAR element-generated miRNA.

**Figure 2:**
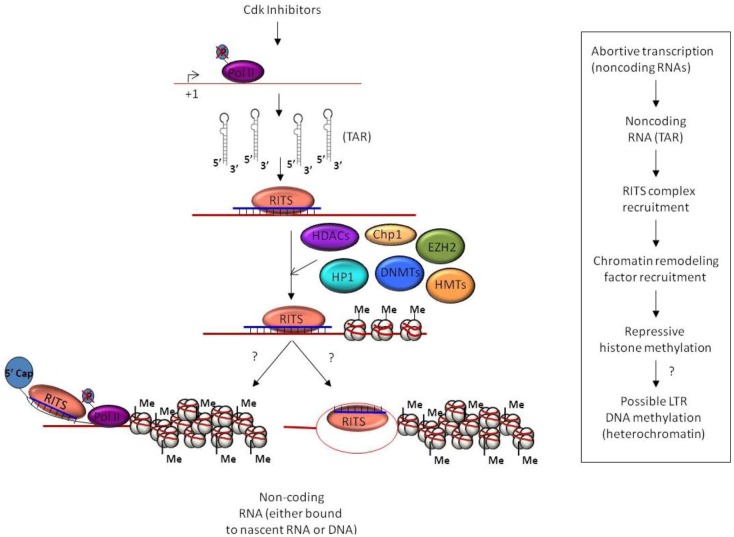
Pol II with hypo-phosphorylated C-terminal domains (CTDs) leads to short abortive transcripts from the integrated HIV-1 LTR, primarily TAR stem-loops. These high copy TAR transcripts are processed into miRNA that assemble with the RITS complex and, subsequently recruit repressive chromatin remodelers. The repressive HDACs, HMTs, DNMTs and other remodelers generate heterochromatin at the LTR and thereby establish viral latency. The use of Cdk inhibitors can increase the levels of un-processive Pol II complexes and, therefore, the levels of nascent TAR transcripts.

Our lab reported that the HIV-1 TAR miRNA prevents cells from undergoing apoptosis in infected and transfected cells, in a Dicer dependent manner [72]. This study indicates that the HIV-1 TAR miRNA is capable of down-regulating cellular gene expression and altering the cellular phenotype. Based on these findings, we’ve postulated that non-processive Pol II complexes at the HIV-1 LTR lead to production of short, TAR containing, RNA hairpin sequences. These hairpins are then subject to cleavage by Dicer resulting in viral miRNA generation. Subsequently, these processed viral miRNAs complex with RISC, which ultimately regulates the expression of several cellular genes through translational inhibition. We reported that the major target of the HIV-1 viral miRNA might be the ERCC1 gene, which is involved in the recognition and repair of DNA damage [83,84]. This finding is in accordance with published reports, which describe HIV-1 up-regulation of the expression of DNA repair proteins [72,85,86]. Moreover, the recruitment of HDAC-1 to the HIV-1 LTR in the presence of either TAR specific siRNA, or TAR RNA, establishes that a TAR-derived miRNA is capable of silencing gene expression through initiating chromatin remodeling and TGS [60]. This suggests the role of viral miRNA in suppression of virus replication and maintenance of viral latency (Figure 2).

In addition to TAR derived miRNA, the 3’ LTR has also been shown to encode a miRNA precursor that can be processed by the RNAi machinery to yield the HIV-1 miR-H1 [87]. The mature miR-H1 has been shown to target the apoptosis antagonizing transcription factor (AATF) and acts as an antagomir against the cellular miR-149. Subsequent to the miR-H1 down-regulation of AATF, Dicer and the prostate apoptosis-4 (PAR-4) gene are also inhibited as AATF regulates both of these genes. Furthermore, due to the indirect down-regulation of Dicer, cellular-wide levels of miRNAs were found to be down-regulated in miR-H1 transfected human mononuclear cells. Moreover, the miR-H1 transfected cells demonstrated an 80% increase in apoptosis as compare to control transfections. From these findings it was postulated that the reduction of the anti-apoptotic AATF gene may contribute to the CD4+ lymphopenia observed with HIV-1 infections. Additionally, the targeting of the host miR-149 was found to be a conserved trait of the virus based on sequencing of clinical HIV-1 isolates, which is beneficial to the virus as miR-149 targets the viral *Vpr* gene.

Another major miRNA encoded by HIV-1 is *nef* derived miRNA. The *nef* gene is located at the 3’ end of the viral genome, which partially overlaps the 3’ LTR. It encodes a 27 to 35 kDa *N-*myristoylated protein, conserved among primate lentiviruses that has been associated with the progression of AIDS [88]. Thought to be involved in viral persistence, Nef also down-regulates MHC-I, which protects infected cells from cytotoxic T-lymphocyte killing. Furthermore, several studies have demonstrated HIV-1 *nef* encodes miRNA. Omoto *et al.* showed the presence of *nef* miRNA from two different strains, specifically the HIV-1 IIIB strain in persistently infected MT-4 T cells and the HIV-1 SF2 strain also in infected MT-4 T cells [8]. These viral miRNAs can block the *trans*-activity of *nef* on cellular genes, as well as inhibit HIV-1 replication. Additionally, *nef*-derived miRNA (miR-N367) can suppress HIV-1 LTR promoter activity through the NRE in the U3 region of the 5′-LTR and *nef* sequences located at the 3′-UTR of targeted regions, demonstrating a promoter interference mechanism by which miR-N367 might reduce HIV-1 transcription [89]. These functions of *nef* via RNAi pathways may contribute to the persistently low pathogenic or latent infection as seen in HIV-infected long term non-progressors [8].

## 3. Epigenetic Regulation and Chromatin Remodeling

Epigenetic regulation and chromatin remodelers play a vital role in viral gene expression as packaging of DNA into chromatin creates a transcriptional barrier [90,91,92,93,94,95,96,97]. The chromatin landscape at the site of the integrated proviral DNA consists of at least five precisely positioned nucleosomes in the region of the chromatinized HIV-1 5’ LTR. The LTR functions as a promoter and contains regulatory elements accountable for viral transcriptional initiation and elongation. Thus, the highly ordered chromatin structure at the 5’ LTR and its modification status dictates the state of active viral replication, latency establishment and reactivation [98,99]. In addition, the integrated viral genome is subject to the same epigenetic regulation as the host genome, chiefly carried out by enzyme complexes that methylate DNA, modify histone tails, or by ATP-dependent enzyme complexes that regulate the interactions of these very same core histones with DNA [99,100]. Due to their importance in transcriptional regulation, many of these epigenetic modifiers play a crucial role in siRNA- and miRNA-mediated TGS.

### 3.1. Histone Acetyltransferases

Histone acetyltransferases (HATs) catalyze the reversible reaction whereby the acetyl group from acetyl-coA is transferred to specific lysine residues (K-) within the N-terminus of histones, neutralizing the positive charge of the tail. This results in hyperacetylation of histone tails, causing a weakened and destabilized histone-DNA or nucleosome-nucleosome interaction, which allows for a more accessible chromatin structure [101,102]. In the context of HIV-1 infection, HATs from the GNAT, MYST, and p300/CBP families have been shown to play an important role [103,104] and will be examined in more detail.

Members of the GNAT family have been shown to play important roles in cell cycle progression, co-activation and elongation by predominantly targeting K14 of H3 or K8 of H4 of both free histones and nucleosomes [102,104]. The largest family of HATs, the MYST family, functions in cell cycle, co-activation, silencing, elongation, damage response, and apoptosis. Targets of these HATs include the H3 and H4 nucleosome subunits [105,106,107]. Members of the third family, p300/CBP, are known to interact with DNA-binding transcriptional factors [108]. These complexes function in a variety of cellular processes including co-activation by preferentially targeting K14 and K18 of histone H3 and K5 and K8 of histone H4 [104]. 

In the scenario of HIV-1 infection, HATs retain normal function but are also recruited to acetylate targets within the viral promoter, resulting in a super-induction of the integrated proviral genome [104,109]. It has been shown that members of the GNAT, MYST, and p300/CBP HAT families target and interact with HIV proteins Tat and Integrase (IN) [103,104,110], and that these interactions may influence localization of the HATs and subsequently alters both host and viral gene expression [109,111]. Ultimately these interactions result in euchromatin formation, the release of transcriptional repressors, and activation of a lytic infection [112].

In regards to HIV-1 associated functionality of HATs, Deng and colleagues demonstrated that the acetylation of Tat by p300/CBP and GCN5 occurred at K50 and K51 [109,113], while p300/CBP also acetylates K28 [104]. Moreover, PCAF has been shown to acetylate Tat at K28 [114], which promotes Tat-CycT1-TAR complex assembly and stimulates transcription elongation [115]. Tat also complexes with p300/CBP, targeting the HATs to specific nucleosomes in close proximity to the viral LTR [104]. Interactions between Tat and p300/CBP and PCAF have also been shown to induce conformational changes in p300/CBP, enhancing the acetylation of H3 and H4 [104]. It has also been shown that the interaction between Tat and TAF_II_250 interferes with the transcription of MHC I genes, aiding in immune evasion [116]. Furthermore, it has been demonstrated that p300 binds and acetylates IN at three residues: K264, K266, K273, enhancing the affinity of IN for DNA and increasing its strand transfer activity [110].

HIV-1 infected cells exhibit attenuated production of the polycistronic miRNA cluster miR-17/19 encoding both miR-17-5p and miR-20a. Within the 3’UTR of the HAT, PCAF, exist four potential target sequences of miR-17-5p and miR-20a and it has been demonstrated that these miRNAs do in fact target the 3’UTR of PCAF to inhibit the translation of mRNA. It has been further shown that miR-17-5p and miR-20a over-expression reduces PCAF expression and HIV-1 production [117]. Thus, viral-mediated down-regulation of miR-17/92 cluster would relieve the repression of PCAF mRNA translation and rescue viral production.

The similarities among HATs in sequence, structure, and enzymatic activity, allow them to serve as simple targets for intervention. Interestingly, pharmacological inhibition of HAT activity has been shown to repress HIV-1 reproduction in SupT1 cells [118]. It has been demonstrated that LTK-14 and garcinol, both of which target p300 and PCAF, reduce proviral expression. Additionally, the microinjection of antibodies against PCAF blocked the transactivation function of Tat [119]. These compounds were found to be nontoxic to T-cells and successful in preventing HIV-1 replication [120]. The inhibition of p300-mediated Tat acetylation has been shown to attenuate proviral expression as well [101]. Given that both p300 and GCN5 specifically bind to IN, the sites involved in this interaction could serve as potential pharmacological targets, ultimately aiming to reduce viral replication [103,110].

### 3.2. Histone Deacetylases

HDACs are the established counterparts to HATs. They function through hydrolytic cleavage and removal of acetyl groups from histones that were added by HATs. This removal generates a hypoacetylated histone tail, which strengthens the DNA-histone or nucleosome-nucleosome interactions and promotes a closed and inaccessible chromatin structure, ultimately repressing transcription [112,121,122]. HDACs are tethered to DNA via interactions with DNA binding proteins, which results in gene silencing [104]. Additionally, the proper functioning of HDACs requires the presence of various co-repressor complexes [122]. 

In the context of HIV-1 infections, HDAC activity promotes the establishment of latency in reservoir cells [104]. Class I HDACs, specifically HDAC1, 2, and 3, have been shown to associate with the HIV LTR, while Class II HDACs-4, 6, and 7 fail to associate with the HIV LTR [123]. Additionally, the recruitment of HDAC1 by YY1 and LSF has been implicated in transcriptional repression of the HIV LTR via the hypoacetylation of nuc-1 [104,124]. Moreover, it has been demonstrated that the binding of HDAC1, in concert with p50 of NF-κB, impairs Pol II recruitment, ultimately preventing transcriptional initiation [125]. HDAC3 also localizes to the HIV-1 LTR in J89GFP cells, a Jurkat based cell line with integrated full length HIV-1 plus green fluorescent protein (GFP) [126], and its inhibition was shown to be necessary for activation of latent HIV-1 [127]. Lastly, HDAC8, which is normally found in the nucleus, has been reported to shift to the cytoplasm in resting CD4+ T cells of HIV-1-positive, aviremic patients on antiretroviral therapy [123].

Another means of TGS includes the targeting of promoter regions by siRNAs, which mediate TGS via formation of heterochromatin [128]. In context of HIV-1 latency, the recruitment of HDAC1 to the HIV-1 LTR in the presence of TAR specific siRNA or TAR RNA was reported. Here, HIV-1 LTR-driven gene expression is inhibited in the presence of an HIV-1 derived miRNA [60]. Interestingly, the induction of TGS by promoter targeted siRNA results in rapidly recruitment of Ago1 and HDAC1, and the increase of the repressive H3K9me2 epigenetic marker at the target site. Additionally, the higher levels of H3K9me2 are also observed both upstream and downstream of the target promoter, particularly at nuc-1, thereby demonstrating histone modifications can extend beyond the targeted promoter [129]. Though it has been shown that the 5’end of the antisense strand of transfected dsRNA can cause specific and sustained activation of targeted genes including E-cadherin, p21 or VEGF, it has also been reported that the introduction dsRNAs specifically targeted to promoter regions induces TGS by triggering histone modification and/or DNA methylation [38]. Both Ago1 and Ago2 have also been implicated with TGS, specifically, playing roles in the RITS complex. Furthermore, it has been proposed that RNA duplexes function as nucleation sites for RITS recruitment and TGS has also been associated with enrichment of dimethylated H3K9me2 and trimethylated H3K27me3, as well as recruitment of HDAC1 [129]. 

In order to modulate HIV-1 activation, the addition of general HDAC inhibitors like TSA, trapoxin, valproic acid, and sodium butyrate to latent cells has been shown to reactivate HIV-1 transcription [104]. Interestingly, HDAC1 inhibition in J89GFP cells fails to reactivate HIV-1 expression, but the further inhibition of HDAC3 by droxinostat reactivates latent HIV-1 in J89GFP cells [127]. Additionally, while HDAC6 does not associate with the LTR, its specific inhibition by tubacin enhances K28 acetylation of Tat and induces Tat-mediated transactivation [130]. Velenzuela-Fernandez and colleagues also report that HDAC6 knockdown increased HIV infectivity and syncytia formation, while over-expression of HDAC6 impaired HIV-1 envelope-dependent infection and cell fusion, yet left the HIV receptor expression and co-distribution unaltered [131]. 

### 3.3. Histone Methyltransferases and Histone Demethyltransferases

In recent years, the role of histone methylation status, as well as the degree of methylation (mono-, di-, or tri-), have been shown to contribute to the modulation of chromatin dynamics and transcriptional regulation [104]. Currently, two groups of histone methyltransferases (HMTs) have been characterized: lysine (PKMTs) and arginine (PRMTs) methyltransferases. These enzymes also methylate non-histone protein targets, and thus have been appropriately renamed protein methyltransferases (PMTs) to reflect this function [132,133,134].

The PKMTs can methylate K4, K9, K27, and K36 on H3 and K20 on H4 through a specific SET-domain containing family of proteins responsible for catalyzing the reversible transfer of a methyl group from S-adenosylmethionine to the lysine residues [133,135]. Alternately, only the methylation of K79 on H3 is not modified through a SET-domain containing protein but rather by the Dot1 family of methyltransferases [136,137]. Lysine residue methylation has been linked to both gene activation and repression, specifically, gene activation has been associated with methylation of H3K4 and H3K36, while gene repression has been linked to H3K9, H3K27, H3K79, and H4K20 [112,133,137,138,139,140,141,142]. Four H3K9 methyltransferases are recognized: Suv39h, G9a, ESET, and GLP, all of which contain the SET-domain. Furthermore, these PKMTs are capable of recruiting various other cellular factors involved in epigenetic regulation [112].

Several lysine specific demethyltransferases have been identified including lysine-specific histone demethylase 1 (LSD1) and jumonji C domain-containing histone demethylation enzyme (JHDM) [143]. Interestingly, Lid2, which serves as a trimethyl H3K4 demethylase, hypomethylates H3K4 resulting in the formation of heterochromatin. It has been shown that Lid2 interacts with other PKMTs like Clr4, and involves the complex containing Dos1/Clr8-Rik1, which also functions in the RNAi pathway. The association of Lid2 with the Rik1 complex enhances methylation of repressive H3K9 epigenetic marker [144].

Several PRMTs have been identified and classified into two types; Type I associated with transcriptional activation and repression, and Type II which is associated with transcriptional repression only [145,146]. Using S-adenosylmethionine as the methyl donor, these enzymes have been shown to mono- or di-methylate arginine residues R2, R8, R17, and R26 on H3, and R3 on H4 [145]. Though previously accepted as an irreversible reaction, the demethylation of arginines is supported by the findings of Cuthbert *et al.*, which support the proposed mechanism of the conversion of methyl-arginine residues to citrulline by peptidylarginine deiminases (PAD), a deimination process [147].

It has been confirmed that the methylation status of proteins modulates the infectivity of HIV-1 [148]. Furthermore, both PKMTs and PRMTs have been implicated in the regulation of viral transcription in HIV-1 infected cells. The PKMTs G9a, Suv39h1, and SETDB1, all members of the SUV39 family, as well as the PRMT1, 5 and 6 have all been associated with either activation or repression of HIV-1 transcription [104,132,149,150,151]. Additionally, the Polycomb repressive complex 2 member EZH2, which catalyzes the formation of repressive H3K27me3, was found to be enriched at the LTR of a latent HIV-1 infected Jurkat cell line [152]. In this regard, PKMTs and PRMTs have been shown to interact with viral proteins and/or HIV-1 associated nucleosomes, resulting in either proviral transcription or latency. 

Along the lines of siRNA-mediated histone methylation, it has been shown that the direct introduction of siRNAs, particularly the antisense strand, specific to the HIV-1 promoter is capable of causing TGS and enhanced histone methylation [153]. In addition to the role of Ago1 in TGS, it has been shown that Ago1 is also required for H3K9me2 [129]. Upon targeted nuclear introduction, siRNA treatment increases H3K9 methylation of targeted promoters [153]. Furthermore, siRNA induced TGS has also been shown to increase EZH2 and associate H3K27me3 levels at a targeted *RASSF1A* promoter [128]. In the same study, levels of EZH2 and Ago1 were also found to be elevated at the promoters of endogenously silenced *MYT1* and *CCR5* indicating a potential link to native TGS mediated by the RNAi machinery.

### 3.4. DNA Methyltransferases

Another key molecular component of epigenetic regulation and chromatin remodeling in mammalian cells is DNA methylation at CpG dinucleotide sites by DNA methyltransferases (DNMTs). It has been well established that basal DNA methylation patterns are dependent upon the methylation status of the K4 and K9 residues of localized H3. Specifically, DNA is found to be methylated in the absence of methyl-H3K4 and the presence of methyl-H3K9 [154,155,156,157]. It has been found that *de novo* methylation of DNA can be carried out by DNMT3A and DNMT3B, which coordinate with the non-catalytic protein DNMT3L [157,158]. In this macromolecular DNMT complex, the amino terminus of DNMT3L has been shown to bind H3 when it is not methylated at the K4 residue, although methylation of H3 at K9 and K27 did not disrupt this intramolecular association. Furthermore, the carboxy terminus of DNMT3L binds to DNMT3A and also drives subsequent dimerization of DNMT3A, thereby establishing the recruitment of the enzymatically active DNMT complex to genomic sites lacking the activating H3K4 methylation modification. 

In addition to the basal establishment of DNA methylation described above, the silencing of active genes requires a complicated sequence of events that also concludes with DNA methylation. In an example of the silencing of the pluripotency gene *Oct3/4* during early embryogenesis, HDACs and repressors proteins first initiate the silencing of the active gene. Next, the HMT G9a is recruited and establishes repressive methyl-H3K9 epigenetic modifications, which allows recruitment of heterochromatin protein 1 (HP1). Finally, DNMT3A and DNMT3B are also recruited by G9a resulting in DNA methylation at the now inactive gene. In total, the sequence of events leads to the establishment of repressive epigenetic markers, the formation of heterochromatin, and long-term gene silencing at the target gene [156].

In order to maintain DNA methylation patterns after DNA synthesis, a DNMT complex must recognize and methylate the unmethylated daughter strand. In this regard, DNMT1 has been shown to associate with the replication complex and, therefore, identified as the key enzyme involved in recognition and conversion of hemi-methylated DNA into double stranded methyl-DNA [156]. 

As will be described in more detail later, several studies have also shown DNMTs to be involved in TGS and the establishment of latency in HIV-1 infected cells. For instance, data from Ishida and colleagues report the hypermethylation of the HIV 5’ LTR in chronically infected latent ACH2 cells, and show that stimulation of these cells with TNF-α attenuated methylation of the 5’-LTR [159]. Furthermore, in the majority of mammalian TGS experiments to date, DNA methylation was found at the targeted promoter sites and in proximal CpG dinucleotides. Moreover, the use of DNMT inhibitors also disrupted the establishment of TGS via promoter targeting siRNAs [7]. However, in contrast to the evidence supporting the involvement of DNMTs in establishing TGS and viral latency, there are also contradicting studies that have found DNA methylation to be lacking in siRNA mediated TGS and latently infected resting CD4+ T cells [160,161]. The conflicting data regarding the importance of DNA methylation in these cellular processes demonstrates that much still needs to be determined in regards to the underlying molecular mechanisms and potential differences between *in vitro* and *in vivo* models.

### 3.5. ATP-Dependent Chromatin Remodelers

In addition to enzymes that methylate DNA or modify core histone proteins, other key chromatin modifiers directly interact with, and manipulate, nucleosomes. For instance, the rapid recognition of covalent modifications on core histone N-terminal tails by members of the yeast SWI/SNF-like (SWItch/Sucrose NonFermentable) complex provides alleviation of chromatin-mediated repression [162,163]. SWI/SNF proteins are part of macromolecular chromatin remodeling complexes (CRCs) that direct ATP-dependent nucleosomal remodeling. They achieve this by employing the energy from ATP hydrolysis to expose nucleosomal-wound DNA via histone-DNA contact disruption, and assemble with the catalytic subunits Brahma-related gene 1 (BRG1), or Brahma (BRM) ATPase, which are ubiquitously expressed in most tissues [164,165]. The SWI/SNF complex is generally accepted as the best-characterized ATP-dependent mammalian CRC [166]. Mammalian SWI/SNF primarily exists in two distinct complexes which are well-defined as Brahma-associated factors (BAF) and polybromo-BRG1-associated factors (PBAF), which exert their differential regulatory function as is suggested by their conserved and unique subunit composition [90,91,167]. 

ATP-dependent CRCs have undergone dynamic modifications themselves over the past decades in terms of classification. In recent years, CRCs have been further categorized into several groups according to their subunit constitution. Interestingly, studies have shown that yeast SWI/SNF acts both as a transcriptional activator and repressor (reviewed in [168]). For example, the specificity of BRG1-containing CRCs such as BAF, PBAF, WINAC, NCoR, NUMAC and mSin3A/HDAC, is supplied by additional subunits, which will confer the CRCs capacity to act as a transcriptional activator or repressor [169,170,171,172]. While ATP-dependent CRCs have yet to be directly tied to mammalian TGS, or the de-repression of genes silenced by TGS, it is likely that interactions occur given the importance of these remodelers in the alteration of chromatin structure. This makes the study of ATP-dependent CRCs in regards to TGS a key, yet unexamined, line of inquiry with significant potential for novel scientific discovery. 

## 4. TGS in Mammalians

Initial evidence of TGS via small ncRNAs in mammals was established using a model system comprised of an elongation factor 1 alpha (EF1α) promoter fused to GFP, which was integrated into the genome of human 293T cells with a feline immunodeficiency virus lentiviral vector. The EF1α-GFP reporter was then silenced by transfection of EF1α promoter targeting siRNA and the silencing mechanism was shown to be induced by DNA methylation using a methyl-DNA sensitive restriction endonuclease digest assay, as well as using DNMT inhibitors [7]. Another early study showed the induced TGS could be achieved with shRNA complementary to the promoter region or up to 23 nucleotides downstream of the transcriptional start site of the RASSF1A tumor suppressor gene. The observed TGS in this experiment also caused low levels of DNA methylation and partial gene silencing in stably transfected HeLa cultures [173].

While the molecular mechanisms involved in mammalian TGS have still not been clearly defined, a set of studies by Kim *et al.* helped to elucidate several of the proteins necessary for this mode of gene silencing [128]. In their studies, mammalian TGS was examined using both transfected siRNA and stably integrated shRNA that targeted either a GFP reporter construct under the control of the *CCR5* promoter or the endogenous *RASSF1A* promoter. Utilizing chromatin immunoprecipitation (ChIP), these experiments again showed enrichment of H3K9me2 at the target promoter of the siRNA, as well as in proximal flanking DNA up to 300-bp downstream of the target promoter. Furthermore, this boost in the H3K9me2 epigenetic marker was found to increase over a 24 hour period post-transfection. Moreover, Ago1 was also enriched at the target promoter and flanking DNA but, unlike the H3K9me2 enrichment, the Ago1 enrichment was transient, peaking at 6 hours post-transfection and then rapidly decreasing over the following 18 hours. It was also found that the siRNA mediated knockdown of Ago1 disrupted both Ago1 and H3K9me2 enrichment at the target promoter following siRNA transfection indicating the role of Ago1 in recruiting siRNA and/or the necessary HMT to the promoter. The involvement of Ago1, as well as Ago2, in mammalian TGS was also found to be required for the formation of the RITS complex in other TGS studies [174,175]. The most recent of these studies showed that HIV-1 promoter targeting siRNAs were found to co-localize with RITS-like component Ago1 in the nucleus and Ago2 at the inner nuclear membrane. This work also indicated the involvement of actin in the transport of this RITS-like complex and showed siRNA specificity in this translocation as scrambled siRNA was retained in the cytoplasm [174].

Beyond Ago1 and Ago2, TAR binding protein 2 (TRBP2) was also found to be enriched at the target *CCR5* promoter after siRNA transfection. Similar to the Ago1 knockdown, the knockdown of TRBP2 via siRNA also blocked subsequent TGS inhibition of the reporter gene mRNA. These data therefore, clearly demonstrated the mechanistic necessity of TRBP2 in RNAi induced TGS [128]. It was also demonstrated that Ago1 was able to co-precipitate with Pol II containing an unphosphorylated C-terminal domain (CTD) after degrading associated RNA, thereby showing a direct protein-protein interaction between these two components. Finally, the histone methyltransferase EZH2, a component of the Polycomb silencing machinery, was also found to be enriched at siRNA targeted endogenous promoters indicating its potential role in the epigenetic modification related to TGS. As EZH2 also boosts H3K27me3 levels, this isoform of H3 was also found to be enriched at the targeted promoters [128]. Furthermore, EZH2 is also able to bind and recruit DNMTs via its homology II domain, thereby providing yet another mechanism by which additional epigenetic silencing may occur [176].

Additionally, experiments targeting the endogenous EF1α promoter with nuclear targeted siRNA also showed an increase in the levels of H3K9me2 and H3K27me3. The observed increase in the H3K9me2 was found up to 720-bp downstream of the targeted siRNA site, demonstrating the spread of the induced epigenetic changes to DNA distal to the target site. Furthermore, a Pol II inhibitor effectively blocked the increase in the TGS associated epigenetic marker, H3K9me2, indicating the requirement for Pol II in siRNA mediated TGS. It was also found that the antisense strand and double stranded siRNA targeting the EF1A promoter also bound to the DNA methyltransferase, DNMT3A. Additional experiments with a HIV-1 LTR reporter cell line further showed that antisense siRNA alone targeting the U3 region of the LTR was sufficient to down-regulate the reporter activity [153].

In addition to the reported host cellular proteins required for induction of TGS in mammalian cell lines, other factors have also been identified in contributing to the efficacy of the TGS by promoter targeting siRNAs. Specifically, one study demonstrated that the ability of a promoter targeted siRNA to generate TGS was also dependent on the rate of transcription of the target gene. In this study, two cancer cell lines that exhibited over a 30-fold difference in progesterone receptor (PR) transcription rates were each transfected with siRNA targeting the PR promoter. In the lower PR expressing cell line, even transfections of up to 100 nM could not induce TGS of the PR gene while concentrations as low as 10 nM were sufficient to generate TGS in the higher PR producing cell line. This study thereby showed that higher expressing genes were more susceptible to siRNA mediated TGS [175]. 

Another key mechanistic question relating to TGS was whether the promoter targeting siRNA formed a duplex with the complementary DNA or nascent mRNA. In this line of inquiry, Han *et al*. showed that alternate RNA transcripts with extended 5’ UTR containing the Pol II promoter region were required for siRNA mediated TGS of *EF1α* and *CCR5* [177]. This study also demonstrated the requirement that the antisense strand of the promoter targeted siRNA form a duplex with the sense strand of the nascent 5’ extended UTR mRNA and not with the comparable DNA strand. This RNA-RNA duplex requirement was verified by targeted degradation of the extended 5’ UTR transcripts which caused significant reductions in the TGS epigenetic markers H3K9me2 and H3K27me3.

While many studies indicated corresponding DNA methylation and histone PTMs with the induction of TGS, others found these epigenetic markers to be unnecessary. Specifically, in challenge of the need for DNA methylation, transfection of a colorectal carcinoma cell line with a pair of siRNAs targeting the promoter of the CDH1 gene caused TGS, as determined by nuclear run-on assays. In this study, no increase of methylation was detected at CpG islands within the targeted promoter, although there was an increase in the levels of H3K9me2, which is associated with inactive promoters. Moreover, a double knock-out of DNMT1 and DNMT3b also did not hinder the TGS of the CDH1 gene in response to the promoter siRNA, contradicting other experimental data using similar double knockouts [161]. Other studies utilizing siRNA that targeted sequences encompassing the transcriptional start sites of several genes also induced TGS without generating local DNA methylation [178]. Similarly, striking differences have been observed between DNA methylation of the 5’ LTR from an *in vitro* model of HIV-1 latency using the ACH2 cell line as compared to latently infected resting CD4+ T cells isolated from aviremic patients on antiretroviral therapy [159,160]. Specifically, the *in vitro* latency model showed significant hypermethylation of the 5’ LTR, while isolated latent HIV-1 infected cells from a group of 11 patients were found to have hypomethylated viral promoters. This conflicting data points to the difficulty in making conclusions regarding *in vivo* systems from data generated from *in vitro* models and is an important consideration for findings regarding mammalian TGS. In addition to questions regarding the necessity of DNA methylation, the requirement for histone modifications in TGS has also been challenged. One study found that the introduction of a HDAC inhibitor did not affect the TGS brought about by the transfection of siRNA targeting the PR promoter [175]. In yet another study, inhibition of the H3K27me3 inducer EZH2 by siRNA resulted in only a low level reduction of subsequently induced TGS [179].

In general, the experiments challenging the importance of the DNA and histone epigenetic modifications in TGS run contrary to the evidence presented in the majority of mammalian TGS studies. However, the questions they pose demonstrate the lack of cohesive theories, or agreement amongst experts, regarding key components in this developing field and indicate the need for a more detailed examination of the underlying molecular mechanisms. Furthermore, while a significant amount of work has been conducted in regards to the utilization of exogenous small ncRNAs, such as siRNA and shRNA, very little evidence links endogenously produced ncRNAs to this important gene expression altering pathway. One study depicted the bidirectional transcription of genes as generating competing sense and antisense strands that could alter gene expression by controlling TGS [40]. In this model, the antisense strand expression could down-regulate the transcription of the complimentary gene through recruitment of Ago1 and the repressive H3K27me3 epigenetic marker to the sense strand promoter. Conversely, reduction of the antisense strand using siRNA could minimize negative epigenetic regulation of the sense strand, thereby boosting sense mRNA transcription. In relation to HIV-1 TGS, numerous studies have identified antisense transcripts and an antisense protein (ASP) produced by HIV-1 over the past several decades [49,77,180,181,182,183,184,185,186,187,188,189,190,191]. Building upon this work, it was recently shown that the U3 region of the 3’ LTR contains a promoter that generates a 2.6 kb antisense RNA (asRNA) with transcriptional termination within the *Env* gene. The promoter of this viral antisense transcript is driven by a NF-кB enhancer site that is responsive to TNFα treatment but not Tat. Furthermore, its over-expression led to repression of HIV-1 replication for up to a month and, conversely, siRNA repression of the asRNA up-regulated HIV-1 replication. While the mechanism by which the asRNA inhibits viral replication was not fully elucidated, it was shown that viral entry and integration were likely not inhibited by asRNA over-expression but that sense strand expression was [192]. These findings could demonstrate that the asRNA transcribed by the HIV-1 provirus could function similarly to the model of competing sense and antisense described earlier. To verify if this viral asRNA does produce TGS, examination of Ago1 and epigenetic markers at the 5’ LTR sense strand promoter need to be examined in the presence of over-expressed or siRNA inhibited viral asRNA. Although this model seems plausible, there is only a relatively small body of evidence supporting it, therefore, the exploration of the interaction between other native mammalian ncRNAs, like miRNAs, and the TGS pathway presents a rich opportunity for novel scientific discovery.

### 4.1. TGS and HIV-1 Infection

A substantial amount of work has been undertaken in the examination of native- and exogenously-induced TGS in HIV-1 infections. Beyond the previously discussed HIV-1 associated TGS experiments, another study used a productive HIV-1 (pNL4.3 strain) infected cell line, MAGIC-5, as a model system. Here, MAGIC-5 was transfected with siRNA complementary to the U3 region of the 5’ LTR at the site of a pair of tandem NF-κB binding sites. This transfection led to RNA-directed DNA methylation (RdDM) and long-term repression of viral transcription, lasting at least 30 days. Other HIV-1 promoter sites were also targeted with varying degrees of viral suppression but the original siRNA construct had the longest lasting viral inhibition. Additionally, the 30 day viral repression greatly surpassed that of a siRNA targeting the Gag mRNA via post-transcriptional gene silencing, which only lasted 7 days [193]. The targeting of the tandem NF-κB promoter site within the 5’ LTR was further shown to be highly specific and could be disrupted by mutations at only 2 to 3 nucleotides in a later shRNA construct. Furthermore, the expression of the shRNA did not induce any TNF-α activated genes that may have indicated TNF-α induction due to TLR3, 7, or 8 recognition of exogenous dsDNA. In that same line of inquiry no PKR activation was observed either. Moreover, the shRNA vector did not down-regulate any off-target genes with NF-κB promoter sequences [194].

In previously indicated studies it was shown that HIV-1 promoter targeting siRNAs were found to co-localize with Ago1 in the nucleus and Ago2 at the inner nuclear membrane. This work also indicated the involvement of actin in the active transport of this RITS-like complex and showed siRNA specificity in the nuclear translocation as scrambled siRNA was retained in the cytoplasm [174].

Another layer of transcriptional silencing dynamics that was touched upon earlier involves HIV-derived miRNA regulation of chromatin remodeling of the viral LTR [60,195]. Here, Dicer-processed HIV-1 TAR-derived miRNA was shown to be capable of regulating viral gene expression and is suggested to potentially repress gene expression through transcriptional silencing via the RITS complex. Similar to the RISC mechanism, the miRNA guides RITS to a complementary region of chromosomal DNA and recruits factors that modify the chromatin structure to the HIV-1 LTR. TAR miRNA has been shown to induce formation of repressive chromatin markers on the HIV-1 LTR, specifically RNAi machinery Ago2 and the histone modifiers Suv39H1 and SETDB1 occupy the integrated LTR and are removed upon heterochromatin abolishment with sub-lethal HDAC inhibitor chronic treatment [195]. The TAR miRNA induced TGS at the HIV-1 LTR could be a key mechanism to establishment of latency in long-lived CD4+ memory T-cells. Conversely, Dicer derived TAR miRNA is less likely to contribute to the pool of latently infected cells of monocyte-macrophage lineage as Dicer was shown to be undetectable in monocytes and repressed in macrophages infected with HIV-1 prior to differentiation [196]. In addition to the generation of viral miRNAs from TAR, sequencing of HIV-1 infected T-lymphocytes has also shown that up to ten percent of the small viral ncRNAs identified were antisense in polarity. Of these a negative polarity transcripts, a majority were derived from the 3’ UTR which could give rise to viral siRNAs that could also lead to TGS and viral latency [49,76].

In addition to naturally occurring TAR miRNA, several Cdk inhibitors such as Flavopiridol and its derivatives, CR8 and CR8#13, have also been shown to boost levels of TAR derived miRNA [195]. The mechanism by which these drugs boost viral miRNAs most likely occurs through blocking transcriptional elongation by Pol II complexes at the HIV-1 LTR. The inhibition of transcriptional elongation in turn generates large quantities of TAR stem-loop RNA that can be subsequently processed by Dicer into miRNA. Additionally, the non-processive Pol II complexes with unphosphorylated CTDs generated by these drugs were also shown to be required for TGS. Therefore, Cdk inhibitors can contribute two-fold in establishing TGS at sites of HIV-1 genome integration by both the generation of TAR stem-loop RNA for miRNA generation and in the seeding of the HIV-1 LTR sites with non-processive Pol II complexes (Figure 2).

## 5. Conclusions

To date, studies on mammalian TGS have begun to decipher a complicated interactive process that involves components of the RNAi machinery and chromatin modifiers. A putative model based on the experiments examined within this review can be found in Figure 1. While the field of mammalian TGS is still in its relative infancy, especially in regards to HIV-1 infections, the potential applications of the findings to date are tantalizing. Specifically, experiments targeting the HIV-1 promoter with siRNA have been shown to establish lasting TGS, which is maintained for over a month *in vitro* with a single treatment. In this regard, the use of siRNAs to induce TGS has already begun to be explored for other varied indications and the specific testing of antisense RNA targeting the HIV-1 promoter in primary CD4+ cells recently demonstrated limited success in reducing viral transcription [197,198]. Additionally, establishment of viral latency through HIV-1 derived miRNAs also serves as a potential point of intervention, as the resultant latent pool could be targeted for reactivation and subsequent killing through the use of anti-retroviral treatments. As additional work in the field of mammalian TGS evolves our understanding of this important molecular pathway, it seems likely that these new insights will help shape the broader view of the inner workings of HIV-1 infections and produce novel methods for the treatment of its associated human epidemic.
